# Experimental Validation of an ITAP Numerical Model and the Effect of Implant Stem Stiffness on Bone Strain Energy

**DOI:** 10.1007/s10439-020-02456-6

**Published:** 2020-01-23

**Authors:** K. Ahmed, R. J. Greene, W. Aston, T. Briggs, C. Pendegrass, M. Moazen, G. Blunn

**Affiliations:** 1 Centre for Biomedical Engineering, Institute of Orthopaedics and Musculo-Skeletal Science, University College London, Stanmore, HA7 4LP UK; 2 Strain Solutions Ltd., Dunston Innovation Centre, Dunston Road, Chesterfield, Derbyshire S41 8NG UK; 3 Bone Tumour Unit and Joint Reconstruction Unit, Royal National Orthopaedic Hospital, Stanmore, HA7 4LP UK; 4 Department of Mechanical Engineering, University College London, London, WC1E 6BT UK; 5 School of Pharmacy and Biomedical Sciences, University of Portsmouth, Portsmouth, PO1 2DT UK

**Keywords:** Amputee biomechanics, Bone density, Bone anchored implants, Digital Image Correlation, Direct skeletal attachment, Finite Element Analysis, Osseointegration, Strain Energy Density, Strain gauge validation, Transfemoral amputees

## Abstract

The Intraosseous Transcutaneous Amputation Prosthesis (ITAP) offers transfemoral amputees an ambulatory method potentially reducing soft tissue complications seen with socket and stump devices. This study validated a finite element (*in silico*) model based on an ITAP design and investigated implant stem stiffness influence on periprosthetic femoral bone strain. Results showed good agreement in the validation of the *in silico* model against the *in vitro* results using uniaxial strain gauges and Digital Image Correlation (DIC). Using Strain Energy Density (SED) thresholds as the stimulus for adaptive bone remodelling, the validated model illustrated that: (a) bone apposition increased and resorption decreased with increasing implant stem flexibility in early stance; (b) bone apposition decreased (mean change = − 9.8%) and resorption increased (mean change = 20.3%) from distal to proximal in most stem stiffness models in early stance. By engineering the flow of force through the implant/bone (e.g. by changing material properties) these results demonstrate how periprosthetic bone remodelling, thus aseptic loosening, can be managed. This paper finds that future implant designs should be optimised for bone strain under a variety of relevant loading conditions using finite element models to maximise the chances of clinical success.

## Introduction

Transfemoral amputees routinely ambulate using a socket (prosthetic cup) and stump (residual limb), this can lead to problems such as skin oedemas, restricted perfusion or tissue necrosis.^33^ Surgical alternatives offered by Skeletally Attached Amputation Prostheses (SAAP) such as the Intraosseous Transcutaneous Amputation Prosthesis (ITAP) channel load through the skeleton. This reduces the problems relating to soft tissue loading and patients cite an improved quality of life with increased prosthetic use.^19^

Inserting relatively stiff implants into bone results in a non-physiological distribution of load, a decrease in periprosthetic bone strain^23^ and culminates in bone loss and aseptic loosening.^25^,^44^ In the mechanostat model^16^ the ‘zone of stress equilibrium’^35^ proposes that a strain-related stimulus holds bone within a homeostatic range by altering the bone mass via adaptive bone remodelling (resorption or apposition). Therefore, managing the stress distribution between the implant and bone, by implant design, could manage aseptic loosening and so prevent removal or replacement surgery.

Adaptive bone remodelling is thought to be governed by the magnitude of the bone strain,^21^ frequency^40^ and rate of loading.^6^ Adaptive bone remodelling simulations using different mechanical stimulus have been compared^34^ and most use change in Strain Energy Density (SED) as the stimulus in both uncemented^22^ and cemented^37^ fixations.

SAAP periprosthetic bone strain measurement is not possible *in vivo* or *in vitro* due to the difficulties in obtaining measurements at the bone implant interface, however finite element (FE) models (*in silico* models) can generate this information. Before reliance on an *in silico* model can be established its accuracy must be assessed.^1^,^41^,^42^ Validated FE proximal femur models^12^ and SAAP FE models in proximal femurs^39^,^44^ are described in the literature, however at the time of writing, there is no study describing a validated *in silico* model of an ITAP in a proximal femur.

The aims of this work were to develop a validated FE SAAP model, based on the design of an ITAP (developed by authors) that has been used in patient clinical trials. Then to use this model to investigate the effects of SAAP implant stem stiffness on periprosthetic bone SED.

## Materials and Methods

### Specimen

A human cadaveric femur from a 59 year old 86 kg male was sourced (Anatomy Gifts Registry, 7522 Connelley Drive Suite M, Hanover, MD 21076, USA) with similar geometry to ITAP patient number 12 in the clinical trial^24^ and then scanned using a Siemens Somatom Definition AS CT scanner (slice thickness = 0.6 mm, pixel spacing = 0.35 mm × 0.35 mm, 512 × 512 matrix). The ‘digital imaging and communications in medicine’ images were interpolated and segmented (Scan IP, Simpleware Synopsis Inc., California, USA) to produce a 3D femur model from which the distal end was resected, leaving 0.201 m (equivalent to ITAP patient 12 residual femur length).

### Experimental Model (*In Vitro*)

#### The SAAP Build

**Figure 1:**
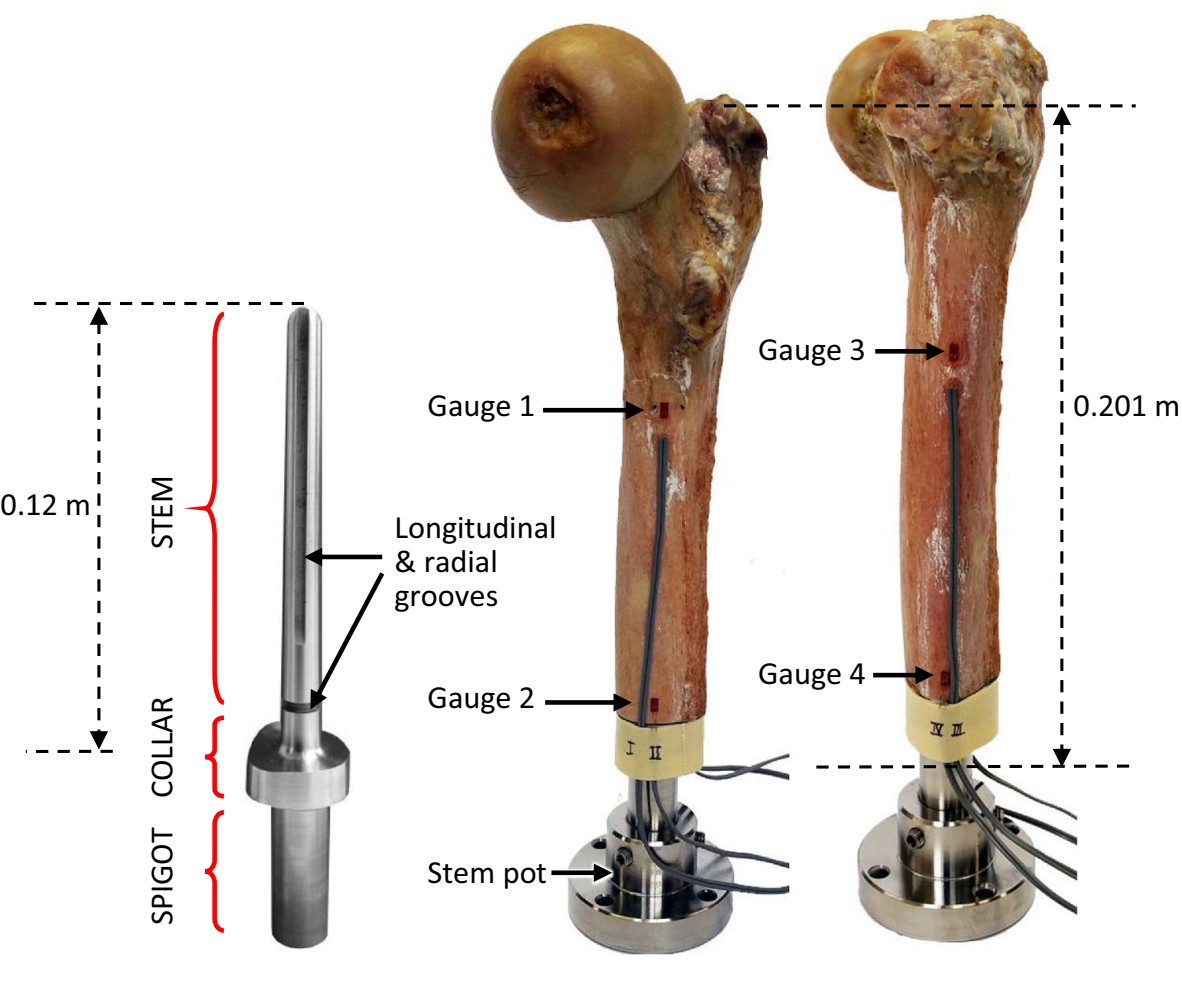
Cadaveric femur photographed medially and laterally with SAAP implanted and potted (also shown seperately). Showing locations of the strain gauges on the medial (left image) and lateral side (right image).

A computer aided design (CAD) model of a SAAP based on the ITAP design was generated (Solidworks, Dassault Systemes, France) and machined (Tritton tooling, Unit 21, Pages Industrial Park, LU7 4TZ, UK) from grade five titanium (TiAl_6_V_4_). The SAAP stem length was 0.12 m with a stem diameter distally of 12 mm narrowing to 9 mm proximally (dimensions equivalent to the ITAP of patient 12) allowing for a minimum of a 1 mm layer of bone cement (polymethylmethacrylate, PMMA). The collar edge shape mirrored the bone osteotomy edge (unlike the ITAP collar which was cylindrical) and the spigot was 18 mm in diameter, the standard size used in all ITAP patients. Four cement grooves (1.5 mm deep, two radially and two longitudinally) were incorporated into the stem design as all cemented ITAP patients were of common design. No grooves were machined onto the collar surface nor was a flange added (*in vivo* these encourage bone ingrowth and soft tissue integration respectively), see Fig. 1.

#### SAAP Implantation into Cadaveric Bone

The bone was stripped of soft tissue, the femoral anteversion angle was measured before the bone’s distal end was resected to leave 0.201 m and squared off using a calcar planer (DePuy Synthes). The fatty marrow and a small amount of cancellous bone on the endosteal surface was removed, the intramedullary (IM) canal was then washed (pulse lavage, Judd Medical, L41100) and dried. A Hardinge cement restrictor was positioned in the IM canal 10 mm proximal to the stem tip and a bone cement mixing system (Cemvac™, DePuy Synthes) was used to deliver the pressurised cement in a retrograde manner. At an appropriate time, the SAAP stem was inserted, and the cement allowed to set. The SAAP spigot was inserted into a stainless-steel (T303) pot and fixed with four 6 mm grub screws, see Fig. 1.

#### Assembly on Load Test Bed

**Figure 2:**
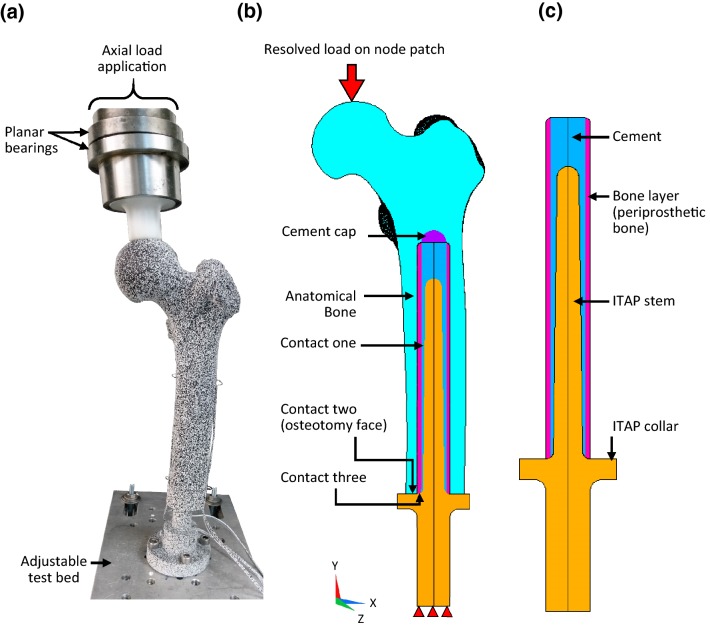
(a) *In vitro* model. (b) Longitudinal section of the *in silico* model assembly showing the bone plug inside the anatomical bone (purple cap = cement material elements, fully bonded to cement layer and anatomical bone). (c) The full bone plug.

The final ‘assembly’ (bone and SAAP) was secured to the load test bed using four M8 bolts at 6.9° femoral adduction, 2.0° flexion and 12.7° anteversion (see assumption one). Axial load was applied through planar bearings at the femoral head on a Zwick Roell, Z005, electrodynamic testing machine (Fig. 2a).

#### Strain Gauges

The periosteal bone surface was cleaned, dried and smoothed with glass paper at four sites; two medially and two laterally for placement of a proximal and distal strain gauge on each. Four uniaxial gauges of 1 mm gauge length (Foil linear goblet gauge 1 mm, 11°C STC, Tokyo Measuring Instruments Laboratory, Japan) were bonded to the bone with a flexible (1.3 GPa) adhesive (Cyanoacrylate-E, Tokyo Measuring Instruments Laboratory, Japan) along the femoral axis (*Y* axis in the global coordinate system), see Fig. 1.

#### Digital Image Correlation (DIC) Set Up

A stereo DIC system consisting of a pair of two megapixel machine vision cameras and ruggedised fixed focal length lenses (Allied Vision Technologies Marlin F-201B, Schneider Kreuznach f1.4/17 mm). The cameras were mounted on a stiff aluminium beam, and this beam mounted on a floor standing tripod. The intrinsic/internal and extrinsic/external calibration parameters of the stereo system were determined by the simultaneous photography of a calibration target containing an array of control points, and this calibration information subsequently used to determine the triaxial location in space of each correlated image speckle subset. The calibration was conducted through a control volume which fully included the whole visible region of the bone, including distance away from the camera system. Typical uncertainty measurements of this system were of the order of one micrometre per measurement point in space.

#### Loading

To settle the specimen a pre-load (100 N) was applied, removed and the system zeroed. Incremental loads were applied as a multiple of body weight (BW) in a range consistent with data from Bergmann *et al.*^3^ in steps up (loading) and down (unloading) to account for bone’s viscoelastic properties from 280.9 N (0.33 BW) to 2949.8 N (3.5 BW). The desired force was maintained for three seconds in which a strain measurement at each gauge and DIC stereo image pairs were recorded from the two cameras and processed using Correlated Solutions Inc. Vic3D 8 software.

### Numerical Model (*In Silico*)

#### Model Development

##### The SAAP Build

The dimensions of the implant were the same as those used for the *in vitro* work except cement grooves were not modelled and the SAAP collar was cylindrical (like the ITAP collar).

##### The Bone Plug Build

A cylindrical *bone plug* was built from second order (20 noded) hexahedral elements (SOLID186) in Ansys Parametric Design Language, ANSYS (v.18.0, Ansys Inc., Pennsylvania, USA). The bone plug comprised: the SAAP, a cement layer and a bone layer (periprosthetic bone). The cement layer at the distal end was 1 mm thick and increased proximally, and the bone layer was uniformly 2 mm thick (Figs. 2b and 2c).

##### Bone Plug Insertion into Anatomical Bone Model

Scan IP was used to create a cylindrical cavity within the anatomical bone model with a larger diameter than the bone’s IM canal. The anatomical bone model was then positioned around the bone plug in a repeatable manner (using the image registration tool). A cement cap was fashioned to join to the top of the cement layer of the bone plug.

#### Material Properties

An idealised orthotropic cortical bone material model (Ashman *et al.*^2^) was selected to represent the bone layer of the plug and the anatomical bone part. Properties were: *EX* = 12.00 GPa, *EY* = 20.00 GPa, *EZ* = 13.40 GPa, *νXY* = 0.22, *νYZ* = 0.35, *νXZ* = 0.38, *GXY* = 5.61 GPa, *GYZ* = 6.23 GPa, *GXZ* = 4.53 GPa (defined in a cylindrical coordinate system where *X* = radial, *Z* = circumferential, *Y* = axial). The bone cement properties were *E* = 2.00 GPa, *ν* = 0.40 and the SAAP was modelled using the ITAP material (TiAl_6_V_4_); *E* = 115.00 GPa, *ν* = 0.30. No cancellous bone was modelled (*E* = Young’s modulus, *ν* = Poisson’s ratio and *G* = shear modulus).

#### Interactions

The cement layer was fully bonded (nodes merged) to the bone layer of the bone plug, the bone layer (second order hexahedral elements) of the bone plug was tied to the anatomical bone (second order tetrahedral elements) with multi point constraint equations, i.e. fully bonded. Three contact surfaces were modelled (Fig. 2b):

1. Between the SAAP stem and the cement layer: contact **one**.
2. Between the anatomical bone (osteotomy face) and the SAAP collar: contact **two**.
3. Between the bone layer (distal face) and the SAAP collar: contact **three**.

Successful ITAP surgery assumes osseointegration (fully bonded surfaces) of the distal bone and ITAP collar, however *in vitro* this is not the case, and the slip between the distal bone parts and the SAAP collar surface was modelled *in silico* by contacts two and three. The model was fully constrained distally (on the face of the SAAP spigot). All contact friction was considered isotropic with a coefficient of 0.30.

#### Boundary Conditions and Load Cases (LC)

Two LC’s were used, one for each part of this study (validation and effects of SAAP stem stiffness):

- LC1 (used for FE model validation): An early stance LC without muscular contribution was applied as a distributed proximal load at the femoral head with the anatomical axis of the femur colinear with the global *Y* axis. An 842.8 N axial load (1.0 BW) was transformed (to account for the femoral orientation *in vitro*) see Table 1. All three contacts described in the *interactions* section were applied to this model.

**Table 1:** Force components in LC1 and LC2.

	FX (lateral (positive)/medial shear)	FY (proximal (positive)/distal force)	FZ (anterior (positive)/posterior shear)
LC1	+ 101.19 N	− 836.19 N	− 29.20 N
LC2	− 804.05 N	− 1957.53 N	− 141.95 N
- LC2 (used with SAAP stem stiffness variations): An early stance LC with an intact musculoskeletal hip joint contact LC^11^ was similarly applied. An early stance LC was transformed using the difference between normal proximal femur (10° flexion, 9°adduction^7^) and SAAP alignment in double legged stance (see assumption one); Table 1. Contact one only was applied; the other contact surfaces were fully bonded.

#### Mesh Convergence

Richardson’s extrapolation^36^ was used to estimate the error in the solution for the bone plug model with normalised element edge lengths of 0.5, 1 and 2. A relative error of < 1% at normalised element edge length of one (0.625 mm) was calculated and so used (full results in appendix Table 3). Bone tetrahedral element edge lengths were matched to 0.625 mm, total element count was 385,080.

#### Measurements

##### Strain Gauge and DIC Node Selection

Surface nodes surrounding the central node corresponding to the centre of each strain gauge *in vitro*, were selected and the mean axial strain was calculated for the validation.

To validate the *in silico* displacement, surface nodes attached to the elements representing the bone DIC visible region were selected. The nodal displacement range falling within a 95% confidence interval (to omit any outlying nodal displacements) was calculated.

##### SAAP Stem Stiffness

The SAAP stem Young’s modulus (115 GPa) was adjusted to 210 GPa and 20 GPa, all other properties were unchanged. The stiffer stem represents biocompatible cobalt chromium (CoCr).^27^ The more flexible stem represents a cellular structured family of metals, additively manufactured from tantalum (Ta) metal.^14^

**Strain:** The SED of a solid is the work done per unit volume to deform a material from a stress free reference state to a loaded state, units are Jm^−3^ (or Pa). SED/ρ thresholds denoting a homeostatic range of 0.0036 Jg^−1^ ≤ bone mass homeostasis ≤ 0.0044 Jg^−1^^30^ were converted to indicate adaptive bone remodelling likelihood. Cross sections were taken at 11 equidistant (1.09 mm) points along the bone layer, Fig. 5a. The percentage of the area in each cross section below, within or above the threshold range was calculated (Adobe Photoshop CS6).

#### Outputs

##### Validation

The outputs from the *in vitro* strain gauges were compared to *in silico* strain in the longitudinal global (*Y*) axis and agreement was measured using the bivariate analysis, Lin’s Concordance Correlation Coefficient (CCC).^28^ The *in vitro* DIC displacement maps were compared (a.) visually and (b.) as a span of displacement (mm) to the corresponding field of view *in silico*, agreement was quantified using CCC.

##### Implant Stem Stiffness

SED results from the *in silico* analysis were computed at each of the 11 cross sections of bone layer in each of the three stem stiffness models. SED in regions below or above the thresholds were considered likely to experience adaptive bone remodelling (resorption or apposition respectively).

#### Sensitivity Analysis

Sensitivity of axial periosteal bone strain at the four gauge sites was investigated in parameters likely to influence a static structural FE analysis. These were bone material and contact properties between parts. A total of 65 models were run.

### Assumptions

#### Assumption one

The assumption that the orientation of the SAAP patient’s femur in early stance being similar to double leg stance has been made in this study. In the absence of joint angle data in the literature for SAAP patients, observations by prosthetists from fluoroscopy results at the RNOH in double leg stance were used.

#### Assumption two

This study assumes that local SED values provide an indication to the bone’s likely initial response to ITAP implantation (local resorption, maintenance or apposition).

## Results

### Sensitivity Analysis

Results were normalised by calculating one standard deviation (SD) as a percentage of the mean strain at each gauge of each model pertaining to the parameter of interest.

- Axial bone strain was sensitive to material property changes in non-linear (contact) models. Gauges one, two and three resulted in sensitivities < 15%, gauge four was 21%.
- Contact types (‘standard’, ‘no separation’, ‘bonded’, ‘rough’ as defined in the ANSYS contact technology manual) had a profound effect on gauges two and four (23% and 88% respectively), but less in gauges one and three (1% and 0.3% respectively).
- The effect of modelling the osteotomy contact surface as 50% bonded resulted in sensitivities < 8% in all gauges apart from gauge four which was 20%.
- Axial bone strain was relatively insensitive to changes in spring stiffness coefficients in rotation and translation between the ITAP spigot and the stem pot (modelling *in vitro* micromotion in the fixing) with sensitivity in all gauges < 10%.
- Axial bone strain sensitivity in the friction models was low (< 5%) in all gauges except gauge four which was 23%.

### Validation

#### Strain Gauge Validation

**Figure 3:**
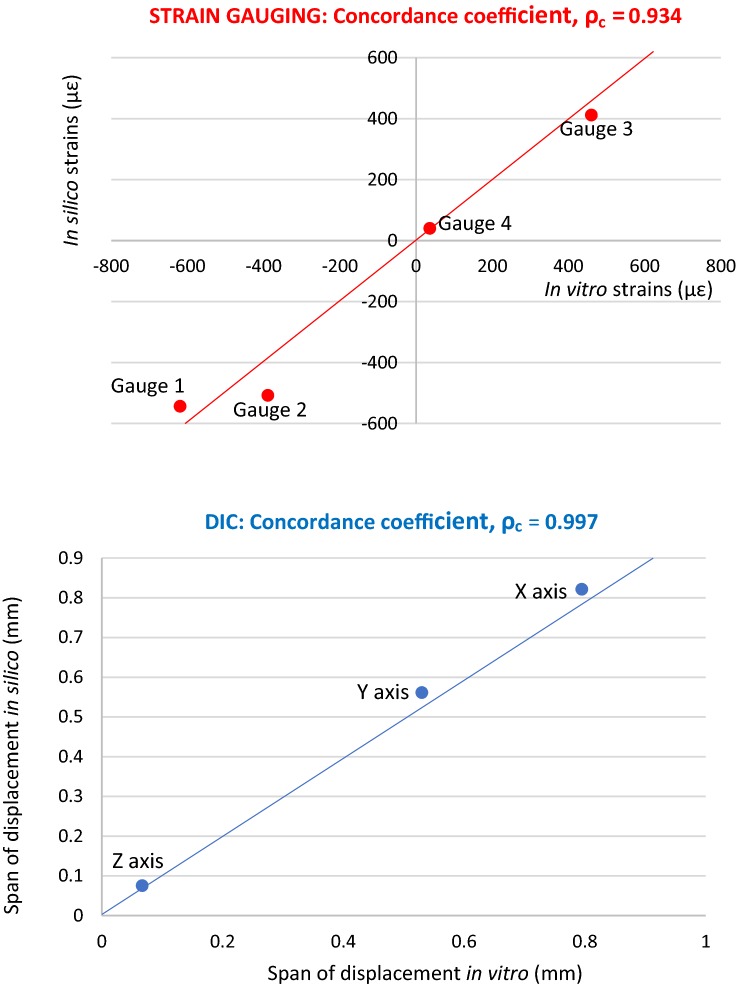
Top = plot *in vitro* against *in silico* strain (με). Bottom = plot *in vitro* against *in silico* displacement (mm).

The CCC produced a correlation *ρ*_c_ = 0.934 between the four mean *in silico* and *in vitro* strain gauge results, Fig. 3. *In silico* strains corresponding to gauge positions one, three and four (error = 12.17%, 10.62% and 9.58% respectively) were closer to their corresponding mean in *in vitro* strains than gauge two (error = 30.79%), Table 2.

**Table 2:** Top = Mean strain (µε) *in vitro* and *in silico* with SD in brackets under LC1. Bottom = displacement (mm) *in vitro* and *in silico* at all gauges/axes under LC1.

Strain (µε)	Gauge 1	Gauge 2	Gauge 3	Gauge 4
Mean *in vitro*	− 619.0 (5.2)	− 388.5 (8.5)	460.5 (2.9)	36.5 (12.7)
Mean *in silico*	− 543.65	− 508.12	411.58	39.997
Error (%)	12.17	30.79	10.62	9.58

Displacement (mm)	*X* axis	*Y* axis	*Z* axis	
*In vitro* span	0.795	0.53	0.067	
*In silico* span	0.821	0.561	0.0749	
Error (%)	3.27	5.85	11.79	

#### DIC Validation

Investigating the *span* of displacement *in vitro* and *in silico*, generated acceptable agreement (Table 2: error = 3.27%, 5.85% and 11.79% for displacement in *X*, *Y* and *Z* respectively) with a CCC of 0.997, Fig. 3.

**Figure 4:**
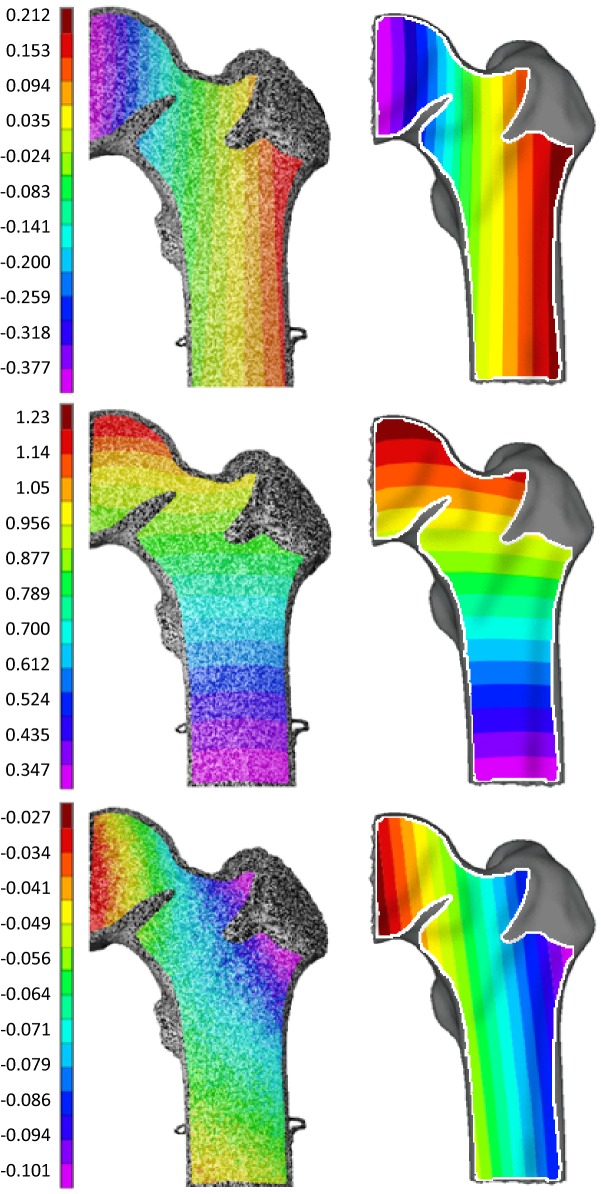
*In vitro* displacement (mm) on the left, *in silico* displacement (mm) on the right. The white line on the *in silico* plots bounds the equivalent DIC camera view area. Top = *Y* axis, middle = *X* axis, bottom = *Z* axis.

Figure 4 illustrates the full field displacement data *in vitro* and *in silico*:

*Y* axis: Displacement along the *Y*-axis was maximum (positive) along the lateral edge and maximum (negative) along the medial edge of the bone DIC record *in silico* and *in vitro*.
*X* axis: The largest displacements *in silico* and *in vitro* along the *X*-axis were proximal and decreased distally.
*Z* axis: Along the *Z*-axis, maximum (negative) displacement was recorded at the greater trochanter *in vitro* and *in silico* and decreased in a diagonal fashion to a minimum at the femoral head *in vitro* and *in silico*.

### SAAP Stem Material Change

**Figure 5:**
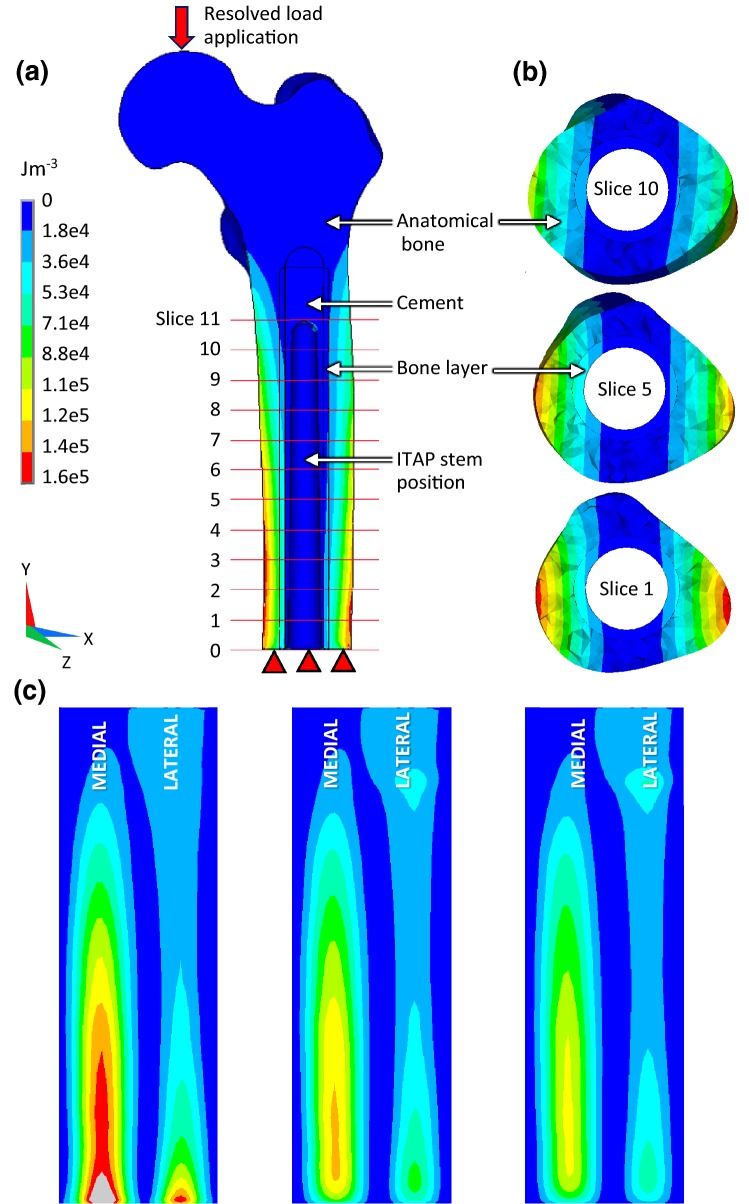
(a) SED (Jm^−3^) in a longitudinal section of the assembly (minus ITAP) showing slice positions 0–11 at 1.09 mm intervals in the periprosthetic bone under LC2 with a 115 GPa stem. (b) SED in transverse section of the bone (anatomical bone + bone layer) at slice locations 1, 5 and 10 under LC2 with a 115 GPa stem. (c) Inner surface of periprosthetic bone layer ‘unwrapped’ showing SED contours in models with a 20 GPa (left), 115 GPa (middle) and 210 GPa (right) stiffness stem.

Figure 5a shows the bone assembly without the SAAP with the location of the 11 periprosthetic bone cross sections (slices). An LC2 load resulted in a mediolateral bending moment about the *Z* axis decreasing moving proximally (Fig. 5b showing the two bone parts only in transverse section).

Figure 5c illustrates the effect on the periprosthetic bone of increasing SAAP stem stiffness (from left to right) under LC2; a reduction in SED medially and laterally is observed. The maximum SED in the more flexible stems in the periosteal bone were 14% and 27% higher when comparing 20 GPa vs. 115 GPa and 115 GPa vs. 210 GPa models respectively. On the periprosthetic bone there was a 50% increase when comparing 20 GPa vs.115 GPa stems and a 13% increase when comparing 115 GPa vs. 210 GPa.

**Figure 6:**
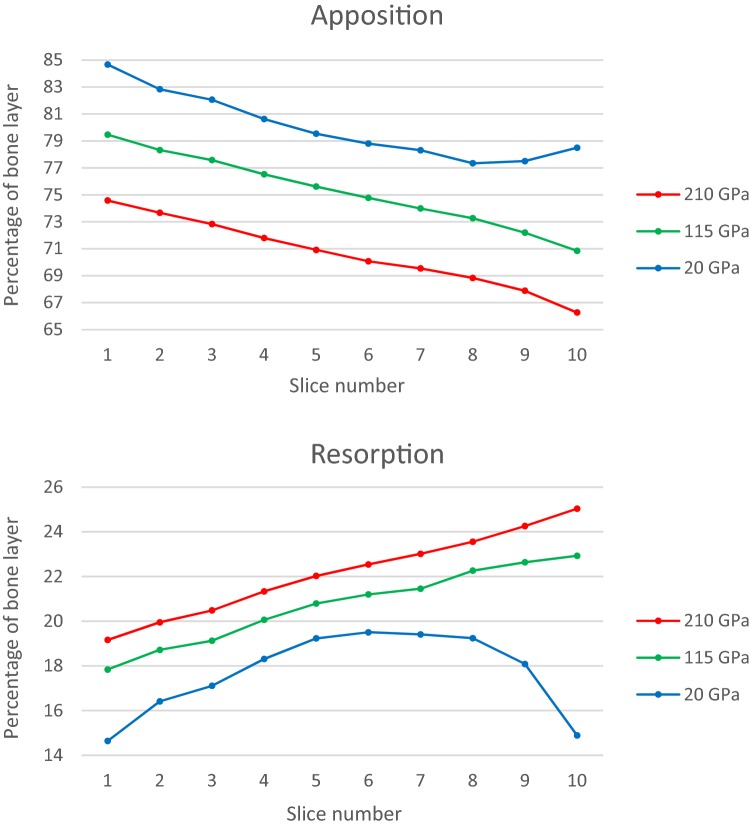
Bone remodelling with respect to SED thresholds along the bone layer (periprosthetic bone) from the first layer proximal to the osteotomy face (slice 1) to the last layer distal to the tip of the ITAP (slice 10) each 1.09 mm apart.

Percentage of total slice area above and below the SED threshold (indicating apposition and resorption respectively) are plotted for slices 1–10 in all stem stiffness models in Fig. 6. Using SED thresholds as the signal for adaptive bone remodelling this shows that (a.) there is more periprosthetic bone apposition in the more flexible stemmed models and (b.) that periprosthetic bone apposition decreases in all stem stiffness models moving proximally. (c.) There is less periprosthetic bone resorption in the more flexible stemmed models and (d.) that periprosthetic bone resorption increases in most stem stiffness models moving proximally. There is an anomaly proximal to slice seven in the 20 GPa stemmed model as resorption area decreases up to slice ten.

## Discussion

### Sensitivity Analysis

The large degree to which bone stiffness and stiffness orientation influenced axial strain results is due to their effect on bone tissue deformation. Most transfemoral amputees present with osteopenic bone through disuse,^17^ the decrease in bone mineral density (*ρ*) is related to Young’s modulus (*E*) by the power law *E* = *aρ*^b^ where *a* and *b* are constants.^20^ Osteopenia inclusion is therefore critical for accurate FE models of SAAP patient assemblies. Cancellous bone was omitted from the model in this study as there was no discernible difference in the axial strain results at any of the gauge sites. This was not unexpected since the most significant effect of LC1 and LC2 loading was to produce a mediolateral bending moment about the *Z* axis in the diaphysis. Due to the bone plug occupying the entire intramedullary canal, the only cancellous bone that was omitted was that in the femoral head. Distal gauge site axial bone strains were highly sensitive to the type and number of contact surfaces employed under LC1 or LC2 suggesting some conflicting convergence criteria. Although three contact surfaces best models the effect of slip *in vitro* at the osteotomy face, caution should be exercised making this choice due to the large error observed *in vitro* axial strain in gauges 2 and 4.

### Validation

A robust discrete point validation corroborated by the full field validation of the FE model has been presented however there were some notable potential sources of validation discrepancy: There appeared to be conflicting convergence criteria when 3 distal contacts were modelled *in silico*; the increased accuracy of the proximally located gauges echoes this finding. Furthermore, discrepancies could have been introduced by visual placement of the uniaxial gauge on the bone being subject to misalignment with respect to the *Y* axis. Additionally, greyscale data from the cadaveric bone CT scan did provide inhomogeneous bone material properties (using density modulus relationships), however these were not employed in either bone part in the *in silico* model. Since the bone plug was housed inside the anatomical bone, both the interface between the outer surface of the plug and the anatomical bone as well as the elements within the anatomical bone would have experienced a step change in elastic modulus. This could have led to a disturbance in the stress distribution between these regions,^29^ potentially resulting in spurious behaviour and so an idealised homogenous cortical bone material (for both bone parts) was selected instead. Lastly, generation of strain information requires local differentiation of the displacement information and inevitably suffers from the introduction of noise and artefacts from the strain calculation algorithm.

Use of single-grid uniaxial strain gauge coupons is an effective method of recording the *in vitro* strain in one direction. It also avoids the use of stacked rosettes where three gauge grids are superposed onto the same measurement location which results in a thick gauge coupon, is difficult to adhere to a curved bone surface and may affect the strain readings. Acceptable *in silico* agreement was observed with a CCC of 0.934; discrete point gauge discrepancies and correlations of this order are similar to those of comparable biomechanical studies.^5^,^31^

#### DIC Validation

Displacement information from the DIC method is of attractive precision and high signal to noise ratio. Since the full surface displacement fields are available from the *in silico* model presented here, a direct comparison has been made between displacement fields, thus avoiding the difficulties associated with the calculation of the second order strain data from the first order displacement information. The displacement field span demonstrates good agreement with slightly larger displacements *in silico* in all axis compared to *in vitro*, with an average error of 7% and a CCC of 0.997. It is possible that the discrepancy between the *in vitro* and *in silico* displacements in the *Z* axis are the result of a torsion that was not calculated by the *in silico* model. A possible reason for this may have been the way that the force was applied or accuracy of the measured angle of anteversion, none the less discrepancies of this magnitude are not unexpected in comparable DIC biomechanical studies.^12^,^18^ Comparison between the experimentally derived displacements and those predicted by simulation would be further improved by the introduction of discrete points of comparison between the two data fields—this will be the subject of future work, with additional full-field mapping of the DIC and FE results.

### Implant Stem Material, SED and Bone Remodelling

Managing aseptic loosening of SAAP due to periprosthetic bone resorption is key to clinical success, as studies using similar endoprostheses have shown.^4^,^8^,^9^ Endosteal resorption will destabilise the implant, conversely if osseointegration and bone growth into the collar can be achieved without radiolucency, then the implant will be stabilised.^8^,^15^ The damage repair theory suggests that when damage from fatigue or impact occur, bone can detect, remove and replace it within resorption cavities.^32^ Immediately post surgically and over time, impact and fatigue damage signals (such as microcracks cutting through the processes of osteocytes^13^ and/or osteocyte apoptosis) will affect the remodelling output as well as the SED remodelling signal. *In silico* models in this study have shown periprosthetic adaptive bone remodelling changes in response to SAAP stem stiffness modification (Figs. 5c and 6).

Since each part of the assembly will carry a portion of the load proportional to its stiffness results were as expected; a higher SED in periprosthetic bone when the stem stiffness was reduced (therefore a larger area of the bone crossed the SED apposition threshold) and vice versa. Furthermore, the distribution of strain energy was greatest distally and decreased proximally (Figs. 5a, 5b, 5c and 6). Summation of the bending moments (Varignon’s theorem) produced from the components of LC2 will deliver this approximate solution.

FX of LC1 is positive whereas in LC2 it becomes negative as the adductor muscles generate the medial forces of early stance.^26^ The value of patient specific load cases, bone models and implant design in predicting regions of adaptive remodelling will be critical for accurate FE modelling of SAAP patient assemblies. To date this has not been a consideration for transfemoral implants but may be important in the design of individualised implants and in the positioning of the external prostheses relative to the spigot.

Obtaining similar strain results to this study in a collared SAAP design, Tomaszewski *et al.*^38^ demonstrated the effect of stem material change on periosteal bone strain. Using experimental and numerical models they showed that the distal and middle gauges and nodes respectively, in three different loading cases, experienced strains 21–29% higher using a more flexible stem. In other SAAP designs with a stiff stem (115 GPa), such as the Osseointegrated Prostheses for the Rehabilitation of Amputees (screw fit), distal bone resorption has been shown clinically and in numerical models.^44^ The inclusion of a SAAP collar in pressfit designs such as the ITAP appears instrumental in managing distal bone strain, hence clinical success.

Manufacture of porous metals is by electron or laser beam sintering a metal powder; the resultant material fatigue limit is usually exceeded due to the nucleation of cracks from pores.^45^ In the case of a fully porous load bearing SAAP stem, especially one that may not be ingrown by bone (this cannot be assumed), further work needs to be undertaken to ascertain the risk of implant fracture. Hypothetically, a porous stem blended into a solid collar and spigot would be the design goal.

In transfemoral amputees muscle groups are removed or transacted and only partly functioning which contributes to osteopenia and remodelling.^17^ Periosteal and endosteal bone resorption will decrease the cortical area and the bone’s resistance to bending and in combination with a decrease in bone density, presents a different material to a stress analysis than the one used in this study. Accordingly, adaptive bone remodelling may produce a different material distribution and a transient analysis using a bone remodelling algorithm^10^,^43^ may be a consideration to monitor the bone change over time.

Using SED as the key indicator for periprosthetic adaptive bone remodelling the value of implant stiffness has been demonstrated. This validated numerical model will allow further studies to be conducted in order to quantify bone remodeling considering variations such as implant material, geometry and fixation type. These encouraging results could mean that future SAAP implant designs should be optimised for bone strain under a variety of relevant loading conditions using FE models to maximise the chances of clinical success.

## Appendix

See Table 3.

**Table 3:** Results of Richardson’s extrapolation for bone plug with a constant grid refinement ratio (*r* = *h*_3_/*h*_2_ = *h*_2_/*h*_1_ = constant) and the observed convergence rate obeying: $p = \frac{{{ \log }\left( {\frac{{f_{3} - f_{2} }}{{f_{2} - f_{1} }}} \right)}}{\log r}$ such that *f.exact*$\approx f_{1} - \frac{{f_{2} - f_{1} }}{{r_{21}^{p} - 1}}$

	Most coarse mesh		Most fine mesh
Normalised element edge length, *h*	2	1	0.5
Maximum stress in *Y* axis (Pa), *f*	1,421,900	1,422,700	1,422,800
Element edge length refinement ratio, *r*		2.000	2.000
Relative error, *e*		0.056%	0.007%
Error to exact solution		0.008%	0.001%
Grid Convergence Index, *GCI*		0.010%	0.001%
95% Confidence interval			
Lower bound		1,422,557.143	1,422,782.143
Upper bound		1,422,842.857	1,422,817.857
Estimate of exact solution, *f.exact*		1,422,814.286	1,422,814.286

## Abbreviations

ITAP: Intraosseous Transcutaneous Amputation Prosthesis
LC: Load case
SAAP: Skeletally Attached Amputation Prostheses
